# A case-control study of factors associated with caesarean sections at health facilities in Kabarole District, Western Uganda, 2016

**DOI:** 10.11604/pamj.2018.29.179.14870

**Published:** 2018-03-27

**Authors:** Jacinta Dusabe, Joseph Akuze, Angela Nakanwagi Kisakye, Benon Kwesiga, Peter Nsubuga, Elizabeth Ekirapa

**Affiliations:** 1 Department of Health Policy, Planning and Management, Makerere University College of Health Sciences School of Public Health, P.O Box 7072, Kampala, Uganda

**Keywords:** Caesarean section, determinants, case-control, Western Uganda

## Abstract

**Introduction:**

World Health Organization estimates that the appropriate caesarean section rates should range from 10% to 15% at the population level. There is limited access and utilisation of caesarean section services in Uganda. This case-control study explored factors associated with caesarean section delivery, focusing on service-related and individual level factors.

**Methods:**

we interviewed 134 cases that had a caesarean section and 134 controls that had a “normal” vaginal delivery. The study was conducted at health facilities in Kabarole district during March to May 2016. Multivariable logistic regression was used to determine individual factors associated with caesarean sections, at a significance level of p < 0.05. Key Informant (KI) data obtained from health workers was analysed using MAXQDA (version 12) software to determine health service factors affecting caesarean section service delivery.

**Results:**

the mean age of the overall sample was 26 years (SD ± 6.5 years). Cases had 5% more women who belonged to the eldest age group (> 35 years) compared to the controls. The factors associated with caesarean section delivery were: having a previous caesarean section delivery (adjusted odds ratio (AOR): 4.5 CI: 2.22-9.0), attendance of four or more ANC visits (AOR: 2.0 CI: 1.04-3.83). Inadequate human resource, medicines and supplies affected access to the service. Misconceptions such as negative branding of women that have caesarean section deliveries as “lazy” reduced its acceptance thus low utilisation of the service.

**Conclusion:**

health system inadequacies and misconceptions about caesarean section delivery contributed to the low access and utilisation of the service.

## Introduction

Caesarean section is one of the most performed surgeries in obstetric practice globally. The World Health Organization (WHO) estimates that the appropriate caesarean section rate should range from 10% to 15% at the population level [1, 2]. Globally, 6.2 million unnecessary caesarean sections are performed and yet 3.18 million additional caesarean sections are needed [1]. There are inadequate caesarean section rates in most developing countries due to limited access to the service and health systems inadequacies while there is adequate or even unnecessary caesarean section use in middle and high-income settings [3, 4]. Studies show that women who have a high level of education and are wealthy were more likely to have a caesarean section [5-7]. However, medical indications account for 77% of caesarean section [3]. Women who have had one birth by caesarean section usually give birth the same way to reduce the risk of uterine rupture; however, a number of studies, show that vaginal births to women with previous deliveries by caesarean section can be performed safely [2, 3, 8, 9]. Older maternal age has also been associated with increased co-morbidities and increased perceived need for caesarean section delivery [4, 5, 10]. Optimum provision of caesarean section services is however hampered by weak delivery systems characterized by inadequate human resource, drugs, supplies and poor infrastructure development [11-16]. Furthermore, there is evidence that misconceptions about health services influence perceptions and attitudes of people in communities to utilise the services [17, 18]. There are few studies and scanty information on factors influencing access and utilisation of caesarean section services in Uganda. The study area has a caesarean section rate of 5.3% [19] at the population level this is low compared to WHO recommended rates [1]. This is an indication of low access and utilisation of the service in the district. This study explored factors associated with caesarean section delivery at health facilities in Kabarole district, Western Uganda. Such knowledge can help planners to set priorities and make appropriate support and resources required to provide caesarean sections available.

## Methods

**Study design and setting:** We conducted a case-control study at health facilities in Kabarole district, Western Uganda, during a 3 month period from March through May 2016. Caesarean section services in the district are offered only at the regional referral hospital and at two health centres in the public sector. While in the private sector, the service is offered at three hospitals. In our study, we included all the three public health facilities that provide caesarean section service and two randomly selected private facilities.

**Case-control definition:** We defined a case as having a caesarean section delivery at the selected health facilities during March, April and May 2016. Controls were women that had a “normal” vaginal delivery at the same health facilities as the cases during the study period. A normal delivery was defined as a spontaneous vaginal delivery without the aid of instrument such as forceps or vacuum extraction. We excluded individuals who had been referred from neighbouring districts in both the case group and control group to avoid selection bias.

**Sample size calculation and sample selection:** A two-stage sampling method was used in this study. At the first stage, we determined the sample size using James Schlesselman's formula for unmatched case-control [20]. The odds ratio associated with exposure that would have sufficient public health importance was hypothesized at 2, using 95% confidence interval and power of 80% a sample size of 134 in the case group and 134 in the control group calculated. At the second stage of sampling, we purposefully included all the three public health facilities that provide caesarean section service in the district and also randomly sampled two private health facilities. We used probability proportionate to sample size sampling (PPS), to distribute the total sample size of 268 amongst the five-selected health facilities. Using the admission list in the maternity wards, study participants that met the inclusion criteria were consecutively sampled. For every case that was selected, a control was recruited.

**Key informant selection:** We also conducted qualitative in-depth interviews with seven purposively selected key informants. These included doctors, midwives and nurses that provide delivery services at the health facilities visited. District health office leaders that included the District Health Officer (DHO) and district in-charge for maternal and child health were also interviewed.

**Variables:** We assessed individual, maternal and health service related factors. Socio-demographic factors included maternal age, education level, marital status, employment status and socio-economic status. Factors related to maternal obstetric and health conditions were parity, multiple births, pre-existing medical conditions and previous caesarean section. Health services related factors were the distance to health facilities measured using geographical distance in kilometres from a place of residence to a health facility, prenatal care consultations (ANC), availability of health infrastructure, human resources, drugs and supplies.

**Data sources, measurement and quality control:** Data were collected in the same way for individuals in the case and control groups. Socio-demographic and maternal information was obtained using face-to-face interviews aided by pre-coded, structured pre-tested questionnaires. Key informant (KI) interviews were also conducted on selected health workers aided by KI guides. The research team made daily visits to the hospitals and health centres to identify women that met the inclusion criteria. The interviews were conducted by trained research assistants with a medical background and fluent in the local languages spoken in the study area. Transcribed interviews were compared against recorded interviews to ensure the quality of qualitative data analysed.

**Data analysis:** After descriptive statistical analysis, bivariable and multivariable analyses were conducted using logistic regression at a significance level of 0.05. We conducted the analysis using STATA statistical package version 12. Crude odds ratios (COR) and adjusted odds ratios (AOR) were obtained with their respective 95% confidence intervals (CI). Covariates that were significant at the bivariable level, with a p-value of less than 0.2 and those determined from literature to be associated with caesarean section were entered in the multivariable stepwise (backward and forward) logistic regression model. Hosmer and Lemeshow's goodness of fit was applied to test the quality model. Previous caesarean section was the most significant variable in the model and was hence taken as the main predictor of caesarean section delivery. Interaction and confounding were tested with the main predictor (i.e. previous caesarean section) using stratified analysis. Confounding was further assessed by comparing the crude odds ratio with the adjusted odds ratio obtained using the Mantel Haenszel. A difference between crude and adjusted odds ratios greater than 10% showed a presence of confounding. Key informant recordings were transcribed and transcripts were uploaded into the qualitative analysis software MAXQDA version 12 and we analysed the data following the six steps of the thematic approach developed by Braun and Clarke [21].

**Ethical approval:** We sought ethical approval from the higher degrees research and ethics committee of the Makerere university school of public health, the District Health Officer (DHO) as well as managers of the respective health facilities where the research was conducted. Interviews were conducted after a written informed consent was obtained from the study participants.

## Results

**Quantitative results:** The mean age of the overall sample was 26 years (standard deviation ± 6.5). Women in the case group were relatively older (> 35 years) compared to those in the control group (i.e. 14% of cases and 9% of controls). Women in the case group had a higher level of education compared to those in the control group (i.e. 52% of cases and 48% of controls had attained primary education) (Table 1).

**Table 1:** Distribution of descriptive characteristics of women studied to assess factors associated with caesarean section at selected health facilities in Kabarole district, Western Uganda (2016)

Variable	Overall(n=268)	Case group(n=134)	Control group(n=134)	
	n (%)	n (%)	n (%)	P value
**Age**				
14-24	142(53)	67(50)	75(56)	0.360
25-34	95(36)	48(36)	47(35)	
35-44	31(12)	19(14)	12(9)	
**Marital status**				
Divorced/separated	17(6)	10(8)	7(5)	0.394
Married	212(79)	108(81)	104(78)	
Unmarried	39(15)	16(12)	23(17)	
**Education level**				
No school	25(9)	13(10)	12(9)	0.699
Primary	134(50)	70(52)	64(48)	
Secondary	85(32)	38(28)	47(35)	
Tertiary	24(9)	13(10)	11(8)	
**Occupation**				
Unemployed	76(28)	47(35)	29(22)	0.015*
Employed	192(72)	87(65)	105(78)	
**Socio-economic status**				
lowest	56(21)	29(22)	27(20)	0.840
Second	51(19)	26(20)	25(19)	
Middle	54(20)	23(17)	31(23)	
Fourth	54(20)	28(21)	26(19)	
Highest	52(20)	27(20)	25(19)	
**Parity**				
0-1	141(53)	65(49)	76(57)	0.416
2-4	93(35)	51(38)	42(31)	
>=5	33(12)	17(13)	16(12)	
**Multiple births**				
No	252(94)	124(93)	128(96)	
Yes	15(6)	9(7)	6(5)	
**Pre-existing medical conditions**				
No	236(89)	115(88)	121(90)	0.512
Yes	29(11)	16(12)	13(10)	
**Previous caesarean section**				
No	196 (73)	77(58)	116(89)	<0.001*
Yes	71(27)	56(42)	15(11)	
**ANC attendance**				
0-3	78(31)	34 (27)	44(34)	0.233
4-6	178 (70)	92(73)	86(66)	
**Distance to Health facility**				
0-5km	134(54)	63(53)	71(55)	0.741
6-100km	114(46)	56(47)	58(45)	

* statistically significant variables

**Bivariable, stratified analysis and multivariable factors associated with caesarean section:** At bivariable analysis, women that had a previous caesarean section were more likely to have a subsequent caesarean section compared to those that did not have a previous caesarean section (COR: 5.6 95% CI: 2.97-10.65). Women that were unemployed were 50% less likely to have a caesarean section compared to those that were employed (COR: 0.5 95%CI: 0.30-0.89) (Table 2). Having a pre-existing medical condition was a confounder thus this variable was included in the final model (Mantel Haenszel AOR: 25.4 CI: 8.33- 77.21). There was no interaction due to age and pre-existing medical condition (Table 3). At multivariable analysis, having a primary caesarean section remained statistically associated with caesarean section (AOR: 4.5 CI: 2.22-9.00). Similarly, women that had four or more prenatal consultations were more likely to have a caesarean section compared to those that had less than four consultations (AOR: 2.0 CI: 1.04-3.83) (Table 4).

**Table 2:** Stratified analysis to assess interaction and confounding of exposure factors associated with caesarean section at health facilities in Kabarole district, western Uganda (2016)

Stratified variable	^a^B-D test (p value)	^b^COR	^c^M-H AOR	[(Crude adjusted)]/crude *100
Age group*(*14-25, 26-44)	0.284	23.1 (7.98-90.16)	22.3 (7.86-63.38)	3.5%
Education status (yes, no)	0.845	23.0 (7.98-90.16)	23.4 (8.13-67.48)	1.7%
Employment status (yes or no)	0.212	23.1 (7.98-90.16)	23.6 (8.06-69.12)	2.2%
Pre existing medical conditions (yes or no)	0.219	22.5 (7.78-88.21)	25.4 (8.33-77.21)	12.9%*

a B-D: Breslow-Day

b COR: crude odds ratio homogeneity test

b M-H AOR: Mantel–Haenszel adjusted odds ratio

* cofounding variable

**Table 3:** Bivariable model (logistic regression) of factors associated with caesarean section at health facilities in Kabarole district, western Uganda (2016)

Variable	COR^a^	95% CI	P value
**Age**			
14-24	1(ref)		
25-34	1.1	0.68-1.92	
35-44	1.8	0.80-3.92	0.184
**Marital status**			
Divorced/separated	1		
Married	0.7	0.27-1.98	
Unmarried	0.5	0.15-1.55	0.189
**Education level**			
No school	1		
Primary	1.0	0.43-2.37	
Secondary	0.7	0.31-1.82	0.330
Tertiary	1.1	0.36-3.35	
**Occupation**			
Employed	1		
Unemployed	0.5	0.30-0.89*	0.015
**Socio-economic status index**			
lowest	1		
Second	1.0	0.45-2.07	
Middle	0.7	0.33-1.47	0.966
Fourth	1.0	0.47-2.12	
Highest	1.0	0.47-2.1.4	
Parity			
0-1	1		
2-4	1.4	0.84-2.40	0.310
>=5	1.2	0.58-2.65	
**Multiple births**			
No	1		
Yes	1.5	0.53-4.48	0.420
**Pre-existing medical conditions**			
No	1		
Yes	1.3	0.60-2.81	0.513
**Previous caesarean section**			
No	1		
Yes	5.6	2.97-10.65*	<0.001
**ANC attendance**			
0-3	1		
4-6	1.4	0.81-2.36	0.234
**Distance to Health facility**			
0-5km	1		
6-100km	1.1	0.67-1.79	0.741

COR^a^ Crude Odds Ratios

* statistically significant variables

**Table 4:** Multivariable model (logistic regression) of factors associated with caesarean section at health facilities, western Uganda (2016)

Variable	AOR^a^	95% CI
**Age**		
14-24	1(ref)	
25-34	1.3	0.62-2.55
35-44	2.3	0.68- 8.02
**Occupation**		
Employed	1	
Unemployed	0.5	0.27- 1.05
**Education level**		
No school	1	
Primary	0.8	0.31- 2.17
Secondary	1.0	0.33- 2.80
Tertiary	1.4	0.37- 5.50
**Parity**		
0-1	1	
2-4	1.2	0.57-2.58
>=5	0.7	0.22-2.42
**Multiple births**		
No	1	
Yes	1.8	0.50- 6.41
**Previous caesarean section**		
No	1	
Yes	4.5	2.22-9.00*
**Pre-existing medical condition**		
No	1	
yes	0.8	0.31-2.20
**ANC attendance**		
0-3	1	
4-6	2.0	1.04- 3.83*
**Distance to Health facility**		
0-5km	1	
6-100km	1.2	0.66- 2.15

### Qualitative results

**Health service factors associated with caesarean section delivery:** Several health service factors perceived to affect caesarean section rates were identified from this study. The key themes that emerged ranged from infrastructure, human resource, drugs and supplies as well as misconceptions about caesarean section delivery.

### Positive factors

**Infrastructure improvements:** Health infrastructure improvements were achieved following an intervention (i.e. Saving Mothers Giving Life) implemented by one of the development partners working in the area. The intervention helped the district refurbish as well as construct functional theatres at lower level health facilities. This was highlighted by one of the health mangers at a lower level health facility who commented: *"The theatre was refurbished by Baylor and equipped with appropriate equipment and we are now able to cater for many mothers" (KI 2)*.

**Human resource recruitment:** A key informant also highlighted that critical health workers were recruited following the same intervention and this increased the capacity to conduct caesarean section at some of the health facilities. This health facility manager talked about the different cadres of health workers that had been recruited at the facility, he asserted: *"A number of doctors, midwives and anaesthetist were recruited this has reduced the work load since we attend to many women daily and have been very few and working in shifts" (KI 4)*.

### Negative factors

**Lack of accommodation for human resource:** Lack of accommodation for critical staff involved in caesarean section service provision and absenteeism of staff at health facilities were some of the problems cited in maintaining adequate human resource capacity as noted by a key informant who said: *"Lack of accommodation for critical staff is the biggest challenges...most of these facilities are in rural areas and health workers spend a lot of time travelling to the work place and are not available during night time to cater for mothers that may need the service" (KI 7)*.

**Inadequate medicines and supplies:** Inadequate medicines and supplies such as anaesthesia, gloves, blood for transfusion, oxygen cylinders were mentioned to be one of the factors affecting provision of caesarean section services. This was raised by one of the health workers who stated: *"Blood sometimes is not available in the facility. We had a mother that got APH(antepartum haemorrhage) so we had to send for blood from the blood bank at the regional referral by the time it got here it was already too late" (KI 1)*. Several other health workers who participated in the study confirmed this view, as the following two quotes illustrate: *"We haven't had oxygen cylinders for some days so haven't been able to carry out Caesarean Section in a while" (KI 3)*.*"Mothers sometimes have to buy gloves as well as IV fluids which is a big challenge since some mothers cannot afford and have to be referred"(KI 6)*.

**Misconceptions about caesarean section:** Misconceptions about caesarean section delivery were reported to create fear among women leading to home deliveries and delays in seeking care. Some of the misconceptions reported by the key informants included disregard of caesarean section as mode of delivery, negative branding of women who have a caesarean section as being lazy. This is reflected in the two quotes below: *"Some women do not know that caesarean section is a mode of delivery and they think failing to deliver normally is a crime" (KI 5)*. *"Women that deliver by caesarean section are branded lazy or unlucky by some of their peers and community members" (KI 3)*.

## Discussion

We assessed factors associated with caesarean section delivery at health facilities in western Uganda and found that having a previous caesarean section delivery, ANC attendance for atleast four times were statistically associated with caesarean section delivery. Inadequate drugs and supplies, inadequate human resource provision, lack of accommodation for critical human resource, misconceptions about caesarean section among women also affected access and utilisation of the service. Infrastrcture improvements and human resource recruitment enhanced caesarean section delivery service provision. Previous caesarean section was statistically significantly associated with current caesarean section delivery. This is similar to findings in other studies in developing countries that found a previous caesarean section to be a very significant predictor for progressive caesarean section deliveries [2, 10, 22]. Women who have had one birth by caesarean section may be given a trial of labour that is carefully monitored. However due to poor monitoring of trial of labour especially at lower level facilities women who had a previous caesarean section subsequently give birth the same way. The risk of uterine rupture and preference for caesarean section among urban women of a high socio-economic status also lead to subsquent caesarean sections [4, 8, 17]. Women that had attended more than four antenatal care consultations were more likely to have a caesarean section delivery than those that attended less than four consultations. This finding is similar to what was observed in earlier studies conducted at health facilities in Brazil and Cameroon respectively that found high numbers of prenatal consultations among women that delivered by caesarean section [4, 23]. Antenatal care is the time when a set of practices and attitudes seeking to promote healthy delivery are applied. Women that have many ANC consultations have been identified as “high-risk” pregnant women so are in constant consultation with the doctors to monitor and prevent adverse outcomes. Prenatal consultations provide an opportunity to assess high-risk pregnant women and to provide counselling related to the mode of delivery [24-26]. However, during this period, health providers can potentially influence decision to implement caesarean section due to the cost benefits of the procedure and fear of legal liabilities in cases of perceived obstetric risks to mother or baby that may result during vaginal delivery. This can lead to unnecessary caesarean sections that are a burden to health systems that work with limited budgets [1, 4].

Maternal age was not found to be significantly associated with caesarean section. This is in contrast to other studies that showed that increase in maternal age was associated with caesarean section delivery due to increased co-morbidities such as hypertensive disorders, diabetes and heart disease among others that increase the risk of caesarean section [4, 22, 23]. This might have been due to the relatively younger age distribution in this study where slightly more than half of the respondents were aged 15 to 24 years with fewer women aged above 35 year. There was inadequate human resource capacity to provide caesarean section service mainly at health centre IV level with only two doctors available, when one was sick or on a leave of absence from work the other was left with a heavy workload. Absenteeism of health workers and lack of accommodation for critical staff were the main factors contributing to the inadequate human resource. Studies carried out in African countries have reported human resource crisis characterised by low motivation, absenteeism and shortages at all levels [11-13]. The Saving Mothers Giving Life intervention, which was a current intervention in the district made improvements in infrastructure development, transport referral and human resource improvements however further improvements and sustainability of such interventions are required for long term impacts on health services provision specifically caesarean section in the area [19, 27]. The findings from our study also showed inadequate medicines and supplies for caesarean section service provision especially at lower level health facilities. This was due to supply chain challenges such as poor forecasting, distribution and misuse of drugs and supplies. Other studies also found that poor distribution and misuse of drugs by health workers in developing countries contributed to stock-outs at health facilities [14-16]. Misconceptions about caesarean section delivery among women in the community affected utilization of the service. Some women thought that failure to deliver normally would make one less of a woman and such women were branded as lazy. Such misconceptions create fear among these women, delay seeking care and subsequently led to low utilization of caesarean section services. This is similar to findings in studies conducted in Western Uganda and Nigeria that found low utilization of maternal services due to traditional beliefs and poor perceptions in the community [17, 18]. Our study was conducted at health facilities thus the case group and control group may not have been very representative of the general population because not all women give birth from health facilities in the area. This may have compromised external validity however all possible measures were undertaken to maintain the internal validity of the study. We recommend that further studies should be carried out in the community.

## Conclusion

Health system inadequacies such as drugs and supplies shortages, human resource inadequacies and misconceptions about caesarean section were the main hindrances to access and utilisation of caesarean section services. Previous caesarean section delivery and attendance of at least four antenatal care sessions were the major predictors for caesarean section delivery. Further investments in providing adequate health workers, ensuring adequate provision of drugs and supplies for caesarean section service are required. In addition, measures to improve the productivity of health workers at health facilities should be put in place such as monitoring and supervision by the health authorities. Women should be encouraged to attend at least four prenatal care visits so that women with predictive factors for a caesarean section can be identified, given counselling to facilitate acceptance of caesarean section and ensure delivery in facilities that can provide caesarean sections or that can give an immediate referral.

### What is known about this topic

- Medical indications for caesarean section such as obstructed labour, cephalopelvic disproportion, malpresentation, fetal conditions among others have widely been studied and are known on this topic.

### What this study adds

- The study assesses individual and health service factors that affect access and utilisation of caesarean section.
